# Structure, Properties and Phase Composition of Composite Materials Based on the System NiTi-TiB_2_

**DOI:** 10.3390/ma15155327

**Published:** 2022-08-02

**Authors:** Vladimir Promakhov, Alexey Matveev, Nikita Schulz, Philip Dronov, Alexander Zhukov, Alexander Vorozhtsov

**Affiliations:** Center for Additive Technologies, National Research Tomsk State University, 634050 Tomsk, Russia; aleksey.9595@mail.ru (A.M.); schulznikita97@gmail.com (N.S.); filipp_dronov93@mail.ru (P.D.); zhuk_77@mail.ru (A.Z.); abv1953@mail.ru (A.V.)

**Keywords:** composite materials, self-propagating high temperature synthesis (SHS), high temperature vacuum sintering, phase composition

## Abstract

This article considers issues pertinent to the research of the phase composition, structure and mechanical properties of materials obtained from powders of composite (Ni-Ti)-TiB2, which have prospective applications in aerospace and automotive industry and engine construction. The starting powder materials (Ni-Ti)-TiB2 were obtained by self-propagating high-temperature synthesis (SHS). Research samples were produced using high-temperature vacuum sintering. It was shown that the use of such materials increases the wettability of the particles and allows the production of composites, the density of which is 95% of the theoretical one. Average particle size was 1.54 µm, average microhardness was 8 GPa, which is an order of magnitude higher than the average microhardness of pure nickel-based and titanium-based alloys, and the ultimate strength values were comparable to those of tungsten-based heavy alloys.

## 1. Introduction

Despite advanced physicomechanical properties of many existing conventional alloys, aerospace, engine manufacturing and automotive industries demand higher operating temperatures for engines and power production units to increase their power efficiency and operating ability [1]. Demand has emerged for materials with more advanced properties, namely hardness, strength, wear resistance, high-temperature operability, oxidation resistance, high crack resistance, etc. The main focus of scientists is on composites that are comprised of ceramic phase particles (TiC, Al_2_O_3_, TiB_2_, WC, SiC, TiN, Cr_3_C_2_, etc.) distributed in a metallic or intermetallic matrix [2,3,4,5]. Composites of such grade possess high mechanical strength, hardness, wear resistance and operating temperatures that are difficult to achieve for conventional metal alloys [6,7,8,9]. In [10] the authors investigated the wear resistance of an automobile brake disc sample fabricated from an Al-SiC composite as compared to a brake disc fabricated from gray cast iron. The composite for the brake disc was prepared from an aluminium melt wherein 20 wt % of silicon carbide particles were introduced (the average particle size was 43 µm) and then the melt was mixed. According to these research findings, the wear resistance of the Al-SiC composite sample is significantly higher than that of the gray cast iron samples. Apart from that, the authors determined that the friction coefficient of the composites was 20% higher than that of the cast iron sample, which translates to an improvement in the braking performance of the composite discs. In [11], arc melting was used to fabricate metal matrix composites from a power mixture of NiAl + 8 wt % of TiB_2_. The composites obtained consisted of an intermetallic NiAl matrix with TiB2 particles distributed in it. The authors showed that adding more than 8 wt % of titanium diboride particles into the initial mixture leads to the formation of three-component Ni_x_Al_y_B_z_ phases. As per the composite structure analysis results, it was found that titanium diboride particles were elongated and they were unevenly distributed in the matrix. The authors determined that titanium diboride particles increase the hardness of the composite versus pure alloy from 455 HV to 555 HV. Walunj G. et al. [12] have shown that in Ni-10 wt % TiC composites fabricated by mechanical alloying of a powder mixture of nickel and titanium carbide with subsequent plasma sintering, nickel matrix grain size decreased from 55 nm to 40 nm as compared to pure nickel. High hardness (350 HV) and compressive strength (1510 MPa) were also recorded. Traditionally, metal–matrix composites are obtained by melting the matrix material with subsequent introduction of reinforcing particles into the melt, or by mixing the initial powders of metals and ceramics with subsequent sintering (vacuum sintering, hot pressing, spark plasma sintering). With these methods, it is possible to fabricate composites comprising a metal matrix wherein reinforcing ceramic particles are distributed. However, use of such methods may lead to recrystallization and agglomeration of ceramic particles as well as formation of an inhomogeneous structure due to low wettability of particles. Therefore, the problem of suppressing the recrystallization and agglomeration of ceramic particles in the course of composite fabrication by melting or sintering arises. A possible solution to this problem lies in the creation of composite metal matrix powders. Particles of such powders consist of a metal matrix with uniformly distributed ceramic inclusions. Using composite powders to obtain dense materials during sintering or introducing powders into the base material melt would increase wettability of ceramic particles by the matrix melt. Therefore, the recrystallization and agglomeration of particles would be reduced, and the density of final materials would increase.

In our previous work [13], we demonstrated successful production of (Ni-Ti)-TiB_2_ metal matrix powders from a NiB-Ti powder mixture by self-propagating high-temperature synthesis (SHS). Research of the microstructure of composite SHS powders has shown that composite particles consist of ceramic particles of titanium diboride uniformly distributed in the intermetallic Ni-Ti matrix. The structure was formed in the course of in situ synthesis supported by exothermic reactions between the initial mixture components. It should be noted that the SH synthesis process is supported by the heat produced by these reactions, thus making it possible to obtain powder materials without external energy input [14,15,16,17]. One of the main disadvantages of the SHS method is high porosity of synthesized materials [18]. This disadvantage does not allow the use of SHS technology to obtain dense materials. In the present work, composite SHS powders were used to obtain materials by high-temperature vacuum sintering.

This research aims to investigate the phase composition, structure and mechanical properties of the materials obtained from composite (Ni-Ti)-TiB_2_ SHS powder by high-temperature vacuum sintering.

## 2. Materials and Methods

### 2.1. Initial Matrials

**Figure 1:**
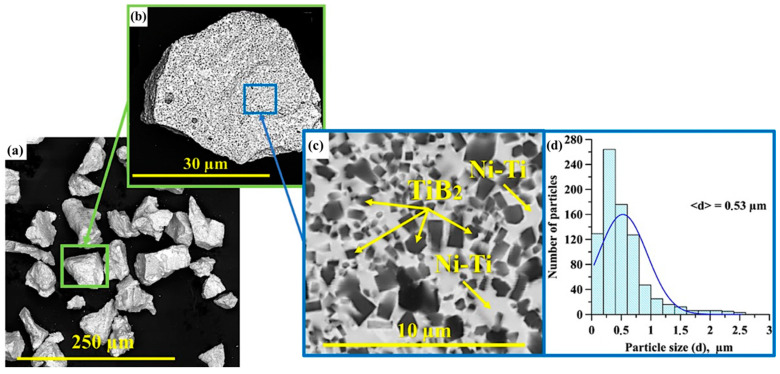
SEM image of NiTi-TiB2 SHS particles (**a**,**b**), SEM image of their structure (**c**) and a histogram of the distribution of TiB2 inclusions by size (**d**).

A NiTi-TiB_2_ composite metal matrix SHS powder was used as the main raw stock for fabricating materials by high-temperature vacuum sintering. The NiTi-TiB_2_ powder was obtained by self-propagating high-temperature synthesis (SHS-powder) from a NiB-Ti powder mixture. The methodology for fabricating metal matrix materials is provided in [13]. Powders of titanium (Ti) and nickel-boron (NiB) were mixed at the stoichiometric ratio 63.5 wt.% NiB + 36.5 wt.% Ti. Samples with a diameter of 23 mm and a mass of 25 g were pressed from the resulting mixture. The obtained samples were placed in an SHS reactor, where the process of SHS synthesis was carried out in an atmosphere of an inert gas (argon). The fabricated SHS materials were ground into powder in an AGO-3 laboratory planetary mill (LLC “GRANAT-E”, St. Petersburg, Russia). The grinding of the obtained materials was carried out in an inert atmosphere of argon. Steel balls with a diameter of 5–15 mm were used as grinding bodies. The mass ratio of the mixture to balls was 1 to 3. The rotational frequency was 15 Hz and the grinding time was 60 min. Particles up to 50 μm in size were separated from the resulting powder using a sieve. Images of NiTi-TiB_2_ SHS particles and their structures obtained by scanning electron microscopy (SEM) (QUANTA 200 3D, FEI Company, Hillsborough, OR, USA) are shown in Figure 1a–c. The size distribution histogram of TiB_2_ inclusions is shown in Figure 1d.

### 2.2. Process of the Fabrication of Composites from NiTi-TiB_2_ Composites by High-Temperature Vacuum Sintering

**Figure 2:**
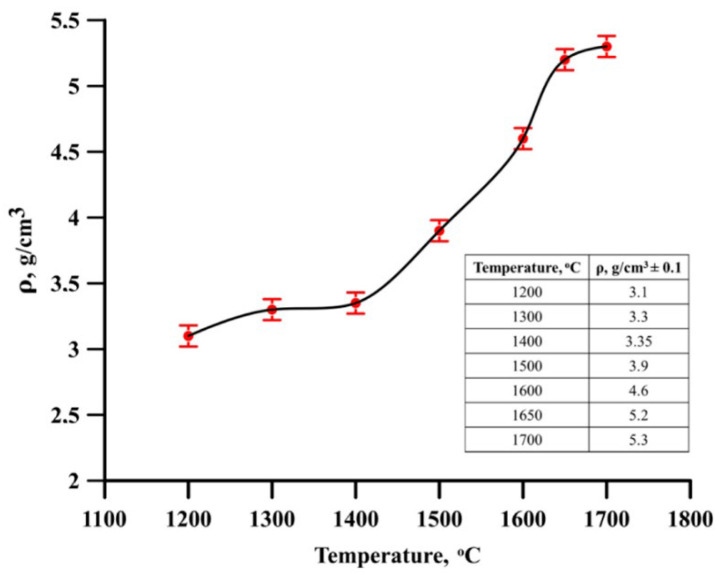
Density vs. sintering temperature curve for the NiTi-TiB_2_ samples.

Rectangular samples were fabricated from NiTi-TiB_2_ SHS powders by single-axis pressing: 30 mm × 20 mm ×10 mm (Figure 2). The pressing pressure was 5 t. The fabricated samples were sintered in a vacuum furnace. The sintering temperature was 1200, 1300, 1400, 1500, 1650 and 1700 °C. For all the samples, the heating time to reach the required sintering temperature was 8 h. The holding time was 1 h for all the samples. The cooling time of the sintered samples was 5 h.

### 2.3. Characterization

The density of the obtained materials was investigated by the Archimedes method. Samples of a parallelepiped shape (10 mm × 5 mm × 3 mm, Length × Width × Height) were obtained from sintered composite materials by electroerosion treatment. Surface of the samples was polished to analyze their phase composition and structure. X-ray diffraction analysis was conducted on a Shimadzu XRD 6000 (Shimadzu Corporation, Kyoto, Japan) diffractometer on CuKα radiation. The phases were identified using the powder diffraction file database (PDF 4). The microstructure of the obtained samples was determined using a QUANTA 200 3D scanning electron microscope (FEI Company, Hillsborough, OR, USA) with energy dispersive spectroscopy (EDS). The size of ceramic particles in the obtained samples was measured by the line intersection method using an SEM image. The microhardness was measured on a Buehler Wilson Micromet 6040 (Buehler LLC, Lake Bluff, IL, USA) hardness meter with a Thixomet Pro image analyzer. The compression tests and three-point bending tests were performed on an Instron universal testing machine (Instron, High Wycombe, UK). Mechanical tests were carried out taking into account an international standard (GOST R ISO 6507-1-2007) as well as a standard approved by Tomsk State University (STO TSU 067-2017).

## 3. Results and Discussion

### 3.1. Sintered Materials Density

Figure 2 shows the density vs sintering temperature curve for the NiTi-TiB_2_ samples.

It was determined that varying the sintering temperature between 1200 and 1700 °C resulted in an increase in the NiTi-TiB_2_ samples density to values between 3.1 ± 0.1 and 5.3 ± 0.1 g/cm^3^. At the same time, the most intense change in density was observed in the temperature range of 1400–1650 °C. The root cause of the density increases at higher sintering temperatures was more intense melting of the matrix material in composite particles which leads to the growth of bridges between them and the facilitation of particle diffusion [19]. The density of NiTi-TiB_2_ materials obtained at a sintering temperature of 1700 °C is 95% of the theoretical one. Based on the data obtained, it can be assumed that the temperature of 1700 °C is the most optimal one for obtaining materials by sintering from the NiTi-TiB_2_ composite powder. Further research was performed on samples obtained at the sintering temperature of 1700 °C and at 1 h holding time.

### 3.2. Phase Composition and Structure of the NiTi-TiB_2_ Composites Fabricated by High-Temperature Vacuum Sintering

**Figure 3:**
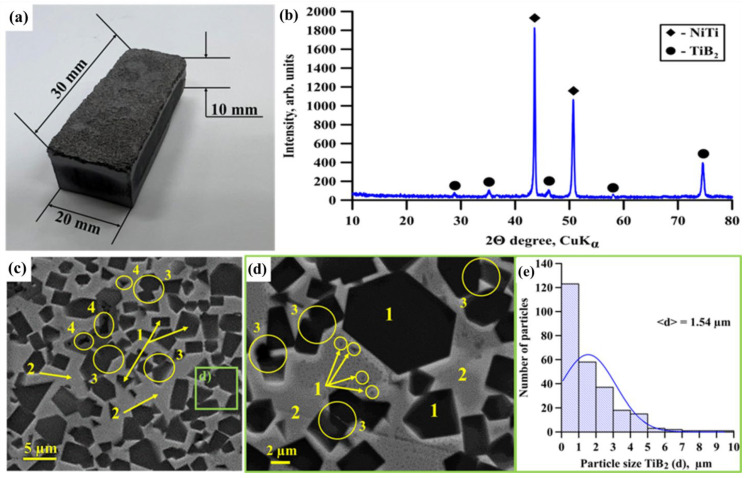
Appearance of sintered NiTi-TiB_2_ composite material (**a**), X-ray diffraction pattern of samples obtained by electroerosion treatment from sintered NiTi-TiB_2_ materials (**b**), SEM images of structure taken from their polished surface: ×1780 (**c**), ×4020 (**d**), TiB_2_ particles (area 1), NiTi intermetallic matrix (area 2), weakly sintered TiB_2_ particles (area 3), pores (area 4), a histogram of the distribution of titanium diboride particles inside the intermetallic NiTi matrix (**e**).

Figure 3a shows the appearance of the NiTi-TiB_2_ composite samples obtained from SHS powders by high-temperature vacuum sintering. X-ray diffraction patterns of samples obtained by electroerosion treatment from NiTi-TiB_2_ sintered materials are shown in Figure 3b. The results of X-ray diffraction analysis are presented in Table 1. Figure 3c–e show SEM images of the structure of material samples taken from their polished surface and a histogram of the distribution of titanium diboride particles inside the NiTi intermetallic matrix.

The X-ray diffraction analysis of the composites uncovered the presence of NiTi (54 wt %) and TiB_2_ (46 wt %) phases. The experimental results were compared to the data in [13], which shows that the main phases of composite SHS particles are NiTi and TiB_2_ as well. However, particles of SHS-produced NiTi-TiB2 powder obtained in [13] also contained traces of the phase of NiTi2 (<6 wt %). Meanwhile, such phase was not present in the composites obtained from our powder by high-temperature sintering. We can suppose that the NiTi_2_ phase was dissolved in the main NiTi matrix material in the course of melting and sintering of composite particles [20]. It can be assumed from the coherent scattering region (CSR) measurement results that the sizes of crystallites in NiTi and TiB_2_ particles are 77 nm and 16 nm, respectively. A comparative analysis of the phase composition of sintered materials and SEM images of their structure with the data of [13] made it possible to establish that the structure of NiTi-TiB_2_ composites obtained by high-temperature vacuum sintering inherits the structure of the particles of the composite powder obtained by self-propagating high-temperature synthesis. The structure of NiTi-TiB_2_ composites is mostly comprised of irregular shaped titanium diboride particles separated from each other (Figure 3c,d, region 1) and uniformly distributed in the intermetallic NiTi matrix (Figure 3c,d, region 2). Apart from that, weakly sintered titanium diboride particles (Figure 3c,d, region 3) were found in the composite structures in amounts that are considerably less than those of separated particles. TiB_2_ particle size was in the range between 0.15 and 10 µm. The average particle size was 1.54 µm. The average size of titanium diboride particles in sintered materials was considerably higher than the average size of those particles in the initial SHS-produced powders (0.57 µm). It is assumed that the formation of weakly sintered particles and an increase in the size is caused by the grinding of SHS products into powder. In the course of grinding, some ceramic inclusions of titanium diboride escaped from the matrix or were located on the surface of particles of SHS-produced powder. After the samples had been shaped by cold pressing, titanium diboride particles were closely interacting with each other. In the course of high-temperature vacuum sintering, these particles adsorbed each other during crystallization, which increased their size and led to the creation of weakly sintered particles. However, the bulk amount of TiB_2_ particles remained in the intermetallic matrix that acted as a dividing layer in the course of sintering. This layer prevented particles from consuming each other, which resulted in the formation of a structure that mostly consisted of separate TiB_2_ particles uniformly distributed in the intermetallic matrix. Therefore, we can make a conclusion that materials with metal matrix structure can be fabricated from SHS powders with a composite metal matrix. Apart from that, a minute number of pores were discovered in the composite structure, which were located in the region of titanium diboride particles, while no pores were found in the matrix material (Figure 3c, region 4). According to the density measurement, it was found that the content of pores was 5%. The presented results confirm the assumption that the temperature equal to 1700 °C is the most optimal for obtaining materials from the NiTi-TiB_2_ composite powder by high-temperature vacuum sintering. In addition, the presented results confirm the highly efficient use of SHS-produced powders with NiTi-TiB_2_ composite metal–matrix particles to obtain materials by high-temperature vacuum sintering. For comparison, in [21] NiAl–Al_2_O_3_ composite materials were obtained by pulsed plasma sintering using aluminum oxide particles. The structure of composites containing 38 vol % Al_2_O_3_ consisted of NiAl particles with aluminum oxide inclusions between them. At the same time, pronounced pores and defects were observed at the boundary between intermetallic particles and ceramic inclusions. In addition, in [22] Al-SiC composite materials were obtained by sintering from a powder mixture of Al-10 vol % SiC (8 and 44 µm). The structure of the resulting composites consisted of an aluminum matrix wherein silicon carbide particles are distributed. Pronounced voids and defects were observed at the particle–matrix interface. In addition, the authors of the work reported that the use of silicon carbide powder with a dispersion of 8 μm leads to agglomeration of ceramic particles and their uneven distribution during sintering. The resulting composites showed low transverse tensile strength (TRS): 49 MPa (<dsic> = 8 µm) and 60 MPa (<d_sic_> = 44 µm). To eliminate these shortcomings, the authors of the work covered silicon carbide particles with a layer of copper using inductively coupled plasma (ICP). The use of the coating made it possible to increase the wettability of ceramic particles, which led to a decrease in pores and defects in the composite structure, as well as an increase in transverse tensile strength (TRS) and normal tensile strength: 231 MPa and 128 MPa, respectively. Thus, the use of ceramic particles pre-coated with a metal layer increases their wettability during sintering, which leads to an improvement in the structure of composite materials and consequently improves their mechanical properties. However, it should be noted that the production of composite powders by self-propagating high-temperature synthesis is based on the fact that ceramic particles (in particular, TiB_2_) are formed during the reaction of the initial components (i.e., in situ) [13]. The obtained ceramic particles are the centers of crystallization of the matrix phase (in particular, NiTi), which prevents their recrystallization and agglomeration processes both in the process of SHS and in the process of sintering, regardless of the size of these particles. In addition, the use of SHS makes it possible to obtain smaller ceramic inclusions compared to conventional sintering using pre-fabricated ceramic particles.

### 3.3. Investigation of the Mechanical Properties of NiTi-TiB_2_ Composites Fabricated by High-Temperature Vacuum Sintering

**Figure 4:**
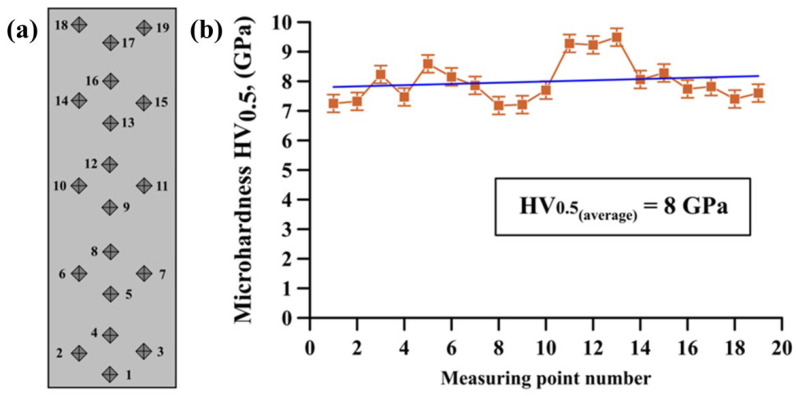
Schematic diagram of the microhardness measuring process in the selected material region (**a**), microhardness measuring results (**b**).

Figure 4a shows a schematic diagram illustrating the microhardness measuring process in the selected region of the material, and the measuring results are provided in Figure 4b and Table 2.

It was established that the distribution of different hardness values in the sample is not always linear and the changes range from 7.2 to 9.5 GPa. Here, minimal hardness values corresponded to the matrix material and maximum values corresponded to large titanium diboride particles with sizes from 5 to 9.5 µm. Average microhardness value was 8 GPa, which is 1.5–2.6-times higher than the average microhardness value for pure alloys based on nickel and titanium as well as heavy alloys based on tungsten [23,24,25]. Increases in the microhardness of NiTi-TiB_2_ samples obtained by high-temperature vacuum sintering are related to high hardness of titanium diboride (25–35 GPa) [26]. These particles provide support for the intermetallic matrix in the composite base, thus boosting average microhardness of the material by means of dispersion strengthening [27].

**Figure 5:**
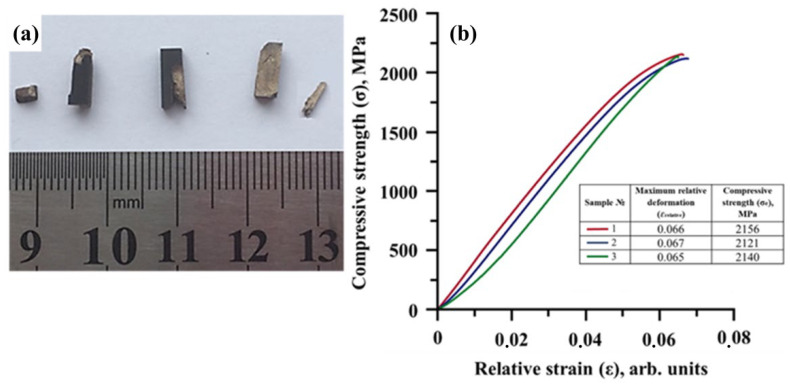
Appearance of NiTi-TiB_2_ samples after the compressive tests (**a**), a stress–deformation diagram obtained during the compressive test (**b**).

Compressive strength tests of the samples were performed on three NiTi-TiB_2_ composite samples obtained by high-temperature vacuum sintering. Figure 5 shows the appearance of NiTi-TiB_2_ samples after the compressive tests and a strain–deformation diagram obtained during the tests.

The results obtained after testing each of the three samples differ insignificantly, which suggests their good correlation and uniform distribution of properties in the material, without prominent gradients. Evidently, this correlation is achieved by the uniform distribution of separate titanium diboride particles in the intermetallic NiTi matrix. The average value of compressive strength limit is 2140 MPa, which is on par with that of heavy tungsten-based alloys (1700–2100 MPa) [25,28] and high-entropy CoCrFeMnNi alloy (1765–2447 MPa) [29].

**Figure 6:**
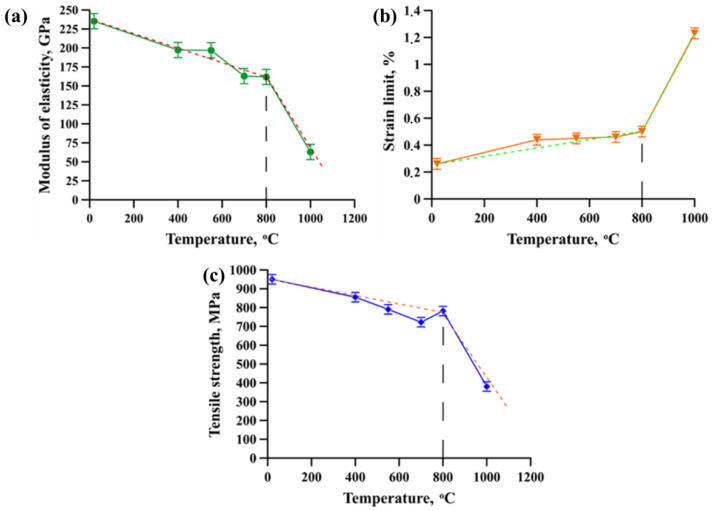
Curves of the modulus of elasticity (**a**), deformation limit (**b**) and ultimate strength (**c**) versus the temperature in the course of bending tests.

As stated above, the main field of application of metal matrix composites is operation in aggressive high-temperature environments. Therefore, the impact of the temperature on the mechanical properties of NiTi-TiB_2_ composites needs to be investigated. Figure 6 shows curves of the modulus of elasticity (a), deformation limit (b) and ultimate strength (c) versus the temperature in the course of bending tests. The measurement results are presented in Table 3.

The curve of the modulus of elasticity versus the testing temperature has steps. A decrease in the modulus of elasticity by 17% is observed in the temperature intervals of 20–400 °C and 550–700 °C. Meanwhile, in the intervals between 400 and 500 °C and between 700 and 800 °C, there is a plateau, which means that the modulus of elasticity is constant or weakly changing. The dependency of the deformation limit versus the testing temperature is linear. Increasing the test temperature from 20 to 400 °C leads to an increase in the deformation limit from 0.26% to 0.44%. In the temperature range between 400 and 800 °C a weak change in the deformation limit (from 0.44% to 0.5%) is observed. The dependency of the ultimate strength versus the testing temperature is linear as well. Increasing the temperature from 20 to 550 °C causes ultimate strength to decrease from 950 to 790 MPa. In the temperature range between 550 and 800 °C, the ultimate strength varied in the range between 722 and 790 MPa. Apart from that, the analysis of the research findings showed that increasing the temperature from 800 °C to 1000 °C leads to a sharp decrease in the ultimate strength and the modulus of elasticity by 2 and 2.6 times, respectively. Here, the decrease in the parameters is accompanied by a sharp increase in the deformation limit by 2.5 times. Therefore, we can suppose that during the three-point bending test, 800 °C is the viscous-brittle transition temperature. Furthermore, it is the critical operating temperature at which the NiTi-TiB_2_ composite retains its mechanical properties (dotted line, Figure 6a–c). The results obtained during the three-point bending tests at high temperatures are comparable with the data on the tests of heavy W-10Cr-0.5Y tungsten alloys obtained by hot isostatic pressing [30]. The authors of the above research works have shown that the bending strength of the alloys at the testing temperature of 800 °C is 725 MPa. The values obtained are in good agreement with the ultimate strength of the NiTi-TiB_2_ composite at the same testing temperature. However, it must be noted that the viscous-brittle transition in tungsten alloys and a sharp decrease in their ultimate strength were observed at the following testing temperatures: 950–1000 °C. Thus, the fabricated composite has advanced physicomechanical properties and it can replace heavier tungsten alloys in the conditions where maximum allowed temperatures may not exceed 800 °C. It should be noted that advanced heat strength values of the NiTi-TiB_2_ composite are achieved by the presence of titanium diboride particles. Thanks to their high melting temperature (3225 °C), high specific heat capacity (43.3 kJ/mol) and thermal conductivity (60–120 W/(m K)), they absorb a part of heat from the intermetallic matrix [31].

Improved strength-related properties of the composite during compression tests and three-point bending tests can be explained by dispersion strengthening that creates a heterogeneous structure and allows for transferring the load from the matrix material to the particle as well as reducing the free path of the propagation of dislocations and cracks [32,33]. Ramakrishnan N [34] demonstrated a composite strengthening model whereby strengthening is achieved by an increase in the average dislocation density caused by the presence of ceramic particles in the matrix. The authors have shown that the highest average dislocation density values are achieved when the size of ceramic particles in the composite does not exceed 3 µm. The research findings by Ramakrishnan N demonstrated a good agreement with the experimental data that are provided in his research paper as well. As stated above, the average size of a titanium diboride particle in the NiTi-TiB_2_ composite was 1.54 µm. In this case, the largest contribution to the particle size distribution is made by particles whose size varies between 0.15 and 3 μm. Thus, based on the results presented in [34], we can suppose that the suggested strengthening model whereby strengthening is caused by an increase in the average density of dislocations can also be applied to the NiTi-TiB_2_ composite produced by high-temperature sintering from composite SHS powders.

## 4. Conclusions

According to the research, the use of composite NiTi-TiB_2_ SHS powders increases the wettability of ceramic particles with the matrix material in the course of high-temperature vacuum sintering. With increased wettability, it is possible to fabricate composites with minimum content of pores (up to 5%) and uniform distribution of separated ceramic TiB_2_ particles in the intermetallic NiTi matrix. The average ceramic particle size was 1.54 µm. The average microhardness was 8 GPa which is 1.5–2.6-times higher than the average microhardness for pure alloys based on nickel and titanium as well as heavy alloys based on tungsten. It was established that the average ultimate strength values obtained by compression tests and three-point bending tests are on par with those of heavy tungsten-based alloys. Meanwhile, the critical temperature at which the NiTi-TiB_2_ composites preserve their mechanical properties is 800 °C. An increase in the strength properties of the composite during the compression tests and three-point bending tests can be explained by the dispersion strengthening mechanism and the strengthening model based on an increase in the average density of dislocations. In the future, it is planned to study the obtained materials for wear resistance. In addition, it is assumed that we will obtain materials of a given shape (cylinder, blades, gear, etc.), as well as testing the obtained products in laboratory and production conditions, in particular fabrication of composite blades for a gas turbine engine.

## Figures and Tables

**Table 1:** Results of the X-ray diffraction analysis of NiTi-TiB_2_ composites fabricated by high-temperature vacuum sintering.

Phases Discovered	Phase Content, Mass %	Lattice Parameters, Å	CSR Size, nm
NiTi	54 ± 3	a = 3.6218 ± 0.0001	77 ± 5
TiB_2_	46 ± 3	a = 2.9468 ± 0.0001 c = 3.1359 ± 0.0001	16 ± 5

**Table 2:** Results of measuring the microhardness of NiTi-TiB_2_ composite materials obtained by high-temperature vacuum sintering.

Measuring Point Number	Microhardness HV_0.5_, (GPa) ± 0.3
1	7.3
2	7.3
3	8.2
4	7.5
5	8.6
6	8.2
7	7.9
8	7.2
9	7.2
10	7.7
11	9.3
12	9.2
13	9.5
14	8
15	8.3
16	7.7
17	7.8
18	7.4
19	7.6

**Table 3:** Measurement results of elastic modulus, strain limit and strength limit as a function of temperature during bending tests.

Temperature, °C	Modulus of Elasticity, (GPa) ± 10	Strain Limit, (%) ± 0.04	Tensile Strength, (MPa) ± 25
20	235	0.26	950
400	197	0.44	855
550	197	0.45	790
700	163	0.46	722
800	162	0.5	781
1000	63	1.23	380

## Data Availability

Not applicable.
